# Interdisciplinary Education Apartment Simulation (IDEAS) Project: An Interdisciplinary Simulation for Transitional Home Care

**DOI:** 10.15766/mep_2374-8265.11111

**Published:** 2021-02-26

**Authors:** Jenna N. Sizemore, Amy Kurowski-Burt, Kimeran Evans, Adam Hoffman, Amy Summers, Gina M. Baugh

**Affiliations:** 1 Assistant Professor, Department of Medicine and Associate Program Director, Internal Medicine Residency Program, West Virginia University School of Medicine; 2 Associate Professor, Division of Occupational Therapy, West Virginia University; 3 Associate Professor of Division of Physical Therapy and Academic Coordinator of Integrated Clinical Education, West Virginia University School of Medicine; 4 Simulation Specialist, David and Jo Ann Shaw Center for Simulation Training and Education for Patient Safety, West Virginia University; 5 Program Specialist of Interprofessional Education and Research Coordinator, David and Jo Ann Shaw Center for Simulation Training and Education for Patient Safety, West Virginia University; 6 Clinical Professor, West Virginia University School of Pharmacy; Director of Interprofessional Education, West Virginia University Health Sciences Center

**Keywords:** Interprofessional Education, Simulation, Transitions of Care, Home-Based Care, Geriatrics

## Abstract

**Introduction:**

Home-based care (HBC) is a valuable tool to provide care to rural, medically underserved populations. By mitigating geographic and transportation barriers for vulnerable populations, HBC is a promising modality of health care delivery. Interprofessional education has become an integral part in undergraduate and professional curricula; however, applications of team-based training in HBC are often missing from curricula. When included, instruction in HBC often utilizes didactic instruction or laboratory experiences, which are discipline-specific and lack a focus on integration of team-based care.

**Methods:**

We implemented a standardized patient (SP) simulation of a posthospital discharge home visit using a team of learners from nursing, physical therapy (PT), occupational therapy (OT), dentistry, pharmacy, and medicine in a simulated home environment. Pre- and postsimulation competencies of interprofessional care were measured using the 20-item Interprofessional Collaborative Competency Attainment Survey (ICCAS).

**Results:**

Throughout the academic years of August 2018 – August 2020, 68 students from nursing, PT, OT, pharmacy, medicine, and dentistry completed a simulated home visit with an SP discharged from a hospital. For all 20 perceived abilities on the ICCAS, learners showed a statistically significant increase in postsurvey measurement. A modest to large (.31 ≤ *r* ≤ .94) effect size was observed in the majority of responses.

**Discussion:**

This SP simulation described a novel, interdisciplinary approach to incorporating HBC into interprofessional curricula.

## Educational Objectives

By the end of this activity, learners will be able to:

1. Describe the roles and responsibilities of an interprofessional team with regards to home-based care (HBC).
2. Develop an effective care plan using an interprofessional team approach for transitions from acute, hospital-based care to the home setting.
3. Demonstrate the use of effective communication among different members of an interprofessional team in HBC.

## Introduction

Americans are living longer; there has been a 30% increase in adults over the age of 65 over the past decade.^1^ As lifespans are increasing, the number of seniors living with chronic medical conditions and needing assistance with activities of daily living and basic household needs is also increasing. Meanwhile, health care professionals are challenged with finding ways to care for patients in their homes.^2^ Home-based care (HBC), defined as any form of assistance provided to a patient directly in the home by family, friends, or community members, utilizes the advice and support from the trained health workers.^3^ HBC has long served an important role in allowing patients to receive care while maintaining their independence of living at home.^3^

As novel strategies to improve patient care and access abound in different practice modalities, HBC has emerged as a desirable option to improve the quality of health care to our rural, underserved patients, including home-bound patients. As HBC works to eliminate geographic and transportation barriers to patients requiring medical services, rural, underserved populations have been projected to have significant benefits from interdisciplinary HBC.^4,5^ Likely due to fewer established HBC organizations, rural residents have lower HBC usage than metropolitan residents while having a significant burden of complex, frail patients.^4^ Improved HBC training programs in areas of suffering from geographic health disparities are needed to increase the supply of HBC providers.^4,5^ However, best practices are varied in representation in undergraduate and graduate health care education. Our simulation sought to expose early learners to the complexities of HBC in the transition of a patient from an academic hospital center to their rural home as a professional skill that can be applied throughout their respective careers.

Simulation activities have been shown to be an effective educational strategy for patient-centered health care education through active learning and reflective observations.^6^ The low-stakes environment of simulation can create an active learning scenario with ample time for feedback and reflection for the student.^6^ In the same vein, interprofessional education (IPE) facilitates learner education by improving interprofessional collaboration and respect for other health care disciplines.^7^ It has been theorized that incorporation of IPE into the early stages of learner training may translate to improving interprofessional collaboration while in professional practice.^7^ Health care training organizations have long proposed increased IPE as a means to reduce medical errors in health care; espousing contact theory as a means to unite different health care groups together to improve relationships.^8^ While there is debate on the effectiveness of IPE in reducing large-scale health care errors, many key competency organizations, including the ACGME, include collaboration as a key feature of graduate training.^8^

However, regardless of the proposed benefits, there remain significant barriers to implementation of IPE programs in practice.^7,9^ The absence of particular professional health programs near training centers or a lack of cross-discipline curriculum structure can affect the integration of IPE into education programs.^7,9^ Scheduling conflicts often arise when coordinating events, and faculty and students' perceptions of IPE can affect the development and integration of educational programs.^7^ A simulation event can offer the opportunity for active learning with several health care disciplines in a collaborative manner, if supported with the right faculty engagement and program resources.

There are numerous simulation activities that focus on discipline-specific goals for transitions to a postacute care setting, but very few focus on interdisciplinary HBC as part of the transitional process. Upon review of our institutional IPE curriculum, we found limited IPE activities involving home and transitional care, and many lacked authenticity and real-world application. As a lack of cross-discipline curriculum structure is a known barrier to incorporation of IPE, an interdisciplinary group of educators developed a comprehensive home-based simulation designed to simulate a home health visit to foster collaboration, education, and respect from learners from medicine, nursing, pharmacy, dentistry, occupational therapy (OT), and physical therapy (PT).^7^ This simulation focused on a postacute hospital care transition with a geriatric patient. This has been an area of focus in health care education with the growing geriatric population and their vulnerability to medical and transitional errors.^10–12^

## Methods

### Development and Personnel

An interprofessional team of professionals from medicine, pharmacy, PT, OT, dentistry, and nursing created this IPE simulation activity describing a home visit to a patient recently discharged from the hospital. Each facilitator contributed to the development of the case (Appendix A) to ensure an accurate depiction of their discipline's focus areas. Case-specific details included improper medical equipment height, placement, and use; environmental features that would increase fall risk; inability to maintain activity limitations based on apartment design and lifestyle; and supplements and foods that might result in medication interactions. This team also served as the activity facilitators throughout the experiences, and therefore no alternate facilitator training was required. When on site, at least one faculty facilitator and one simulation specialist guided the students through the experience.

Simulations were coordinated through West Virginia University's David and Jo Ann Shaw Center for Simulation Training and Education for Patient Safety (WV STEPS). The standardized patient (SP) was selected based on demographic characteristics, including age and prior training for the portrayal of a geriatric patient. One SP was trained on the case and the specific learning objectives prior to the first session by WV STEPS; the trained SP was utilized for all scenarios to maintain consistency in the simulation. However, as our simulation center has a well-established SP program, there was an extensive pool of potential substitute SPs if needed. The SP received moulage to mimic sternal surgery with scar tissue and dressing placement to enhance the authenticity of the scenario.

All students participated on a voluntarily basis; participation was not graded and had no effect on class standing. Students were recruited via email communication by third-party personnel from our simulation center. As participation was voluntary, the learners consisted of different levels of training. The medicine cohort consisted of learners from the second, third, and fourth years of medical school, as well as one team composed of resident physicians in their postgraduate first year. Pharmacy participants were in their third year of the doctor of pharmacy curriculum. PT students were in their second graduate year. OT learners were in the second or third year of education. Undergraduate nursing students were in their third year of education. Fourth-year dentistry students participated in the simulation. Parking validation was provided to the students if needed, which was granted by university housing where the simulation was conducted. Students were not required to pay for the experience.

The interprofessional teams were designed to favor those most involved in home health services. Professions were selected based on schools within our respective health sciences center campus that had previously participated through our institutional IPE program. Notably, learners from the profession of social work were not included as they were housed on a different campus of our academic center. Dentistry, while not traditionally a component of HBC, participated based on their proximity of the health science center campus and faculty interest in the program.

### Environment and Equipment

The home visit was simulated in an on-campus, student residence apartment model room, for which use was granted by the university. Simulation personnel and faculty facilitators prepped the apartment with specific items related to the patient case (e.g., wheeled walker, raised toilet seat, various foods and beverages, medications; Appendix B). Such items were added to the simulation to highlight potential in-home dangers for patients with the numerous, new medications prescribed, and activity limitations due to sternal surgery after discharge. The small groups would interview the patient in the apartment and then walk the environment to discover safety hazards. This allowed each respective profession to find areas that may inadvertently cause the patient harm that they could communicate to the group as a whole. All small-group sessions were recorded with student permission. Approval was granted by our institutional review board. Facilitators were able to watch the scenario unfold, in real time, remotely in a separate room through video streaming.

### Implementation

Participants included students from nursing, medicine, dentistry, pharmacy, OT, and PT throughout the academic years of August 2018 – August 2020. Students from the representative disciplines performed the simulation in small groups of four to 10 individuals. Each session did not have the same combination of disciplines, but always included at least two different learners from a different health profession. The sessions were conducted using sequential small-group sessions over the course of 3 hours. The initial hour was used by the faculty team for patient and home preparation, while each IPE student group was allotted 1 hour for prebrief, simulation completion, and the debrief.

The interprofessional group of students were prebriefed in a conference room adjacent to the apartment about the case details using the discharge summary (Appendix C), introduced to all representative professions, and informed that they would conduct a patient interview and assessment in their home (15 minutes). The student group would then strategize a plan for implementation of the interview and assessment and identified leaders for the process (10 minutes). Generally, the students' interview and assessment with the SP consisted of past medical history, current and past level of functioning, minimal physical assessment of ability (i.e., functional mobility, transfers), medication assessment and discussions, and goal setting. Different groups developed different strategies as to how to conduct the visit, but often nursing, PT, and OT would take the lead in the interview as these are the common HBC practitioners through current home-health services.

After the interview and assessment, the students walked through the apartment to observe the home living situation and identify any environmental (e.g., uneven wheeled walker, clutter in the bedroom) or medical (e.g., alcohol-based mouthwash, inadequate storage of medications) hazards. Lastly, the group formulated a plan to help aid in the improvement of the patient's health after discharge to the home alone. As a team with the SP, the students discussed specific areas that could help to mitigate the potential for patient harm based on their findings. Following the SP discussion with comments and suggestions from the SP, learners had the opportunity to share their personal reflections on team communication, as well what they learned regarding the roles of other professions. A formal debriefing session was held with the learners and at least one faculty facilitator and the simulation specialist who conducted the prebriefing. The simulation encounter was performed over 30–45 minutes. Two separate simulation encounters were performed sequentially by two separate small groups. Depending on the efficiency of the small groups, the first group was debriefed while the second group was performing the encounter.

### Debriefing

The debriefing focused on the interprofessional aspects of the encounter including professional roles and responsibilities, teamwork, and IPE communications. The session typically ranged from 30–60 minutes in length and was done using open-ended questions and a few debriefing techniques (Appendix D).

### Assessment

At the end of the session the 20-item Interprofessional Collaborative Competencies Attainment Survey (ICCAS)^13^ was administered to gain a retrospective pre- and postanalysis of student behaviors regarding IPE. The ICCAS seeks to assess behaviors related to collaborative patient care by measuring changes in participants' perceptions after an IPE event in communication, collaboration, responsibilities, conflict resolution, and team functioning.^13^ All items are rated on a 7-point scale (1 = *strongly disagree*, 2 = *moderately disagree,* 3 = *slightly disagree*, 4 = *neutral*, 5 = *slightly agree,* 6 = *moderately agree,* 7 = *strongly agree,* na = *not applicable).*

Retrospective pre- and postsurvey mean responses were recorded and matched using LearningSpace software (CAE Healthcare) by our simulation center data specialist to ensure anonymity of responses. Statistical analysis was performed using a paired sample *t* test. A *p* value < .05 was used to determine statistical significance, which corresponded to an absolute value greater than 1.96 from the *t* statistic. The Pearson correlation coefficient (*r*) was used to determine effect size of the simulation. All student responses were collected anonymously and independently coded by a data specialist through WV STEPS to ensure anonymity from the researchers.

## Results

Over the course of 2 years, eleven interprofessional teams completed the simulation with a total of 68 student participants. The makeup of learners from the respective professions were as follows: medicine (*n* = 9, 13%), nursing (*n* = 7, 10%), OT (*n* = 14, 21%), PT (*n* = 15, 22%), pharmacy (*n* = 14, 21%), dentistry, (*n* = 9, 13%). The disciplinary composition of teams would vary per simulation given the voluntary nature of the participants. Five teams that completed the simulation included all six specialties. All teams included a representative from OT and pharmacy. Nursing was represented on seven of the 11 teams, and medicine was represented on nine of the 11 teams. PT participated in seven of the 11 teams as well. Due to scheduling, some teams had more than one representative from a respective discipline.

Of students who participated, 68 (100%) completed the ICCAS. Retrospective pre- and postsurvey mean responses are shown in the Table. All 20 presurvey mean responses were above the scale midpoint of 4 out of 7, ranging from a low of *M* = 4.6 (*SD* = 1.7) to a high of *M* = 5.9 (*SD* = 1.1), showing that students felt comfortable with IPE behaviors prior to the simulation. Postsurvey responses showed a statistically significant increase in mean responses for all 20 items on the postsurvey ranging from a low of *M* = 6.1 (*SD* = 1.0) to a high of *M* = 6.8 (*SD* = 0.5). In addition the postsimulation effect size was modest to large (.31 ≤ *r* ≤ .94) with the exception of the third survey question “able to express my ideas and concerns without being judgmental” (*r* = .09).^13^ Most notably, our participants showed increases in self-reported confidence in their ability to promote communication as a member of an IPE team (*M*_pre_ = 5.5, *M*_post_ = 6.5), their understanding of the abilities and contributions of other team members (*M*_pre_ = 4.8, *M*_post_ = 6.5), and to develop an effective care plan with other health care professionals (*M*_pre_ = 4.7, *M*_post_ = 6.3).

**Table.**
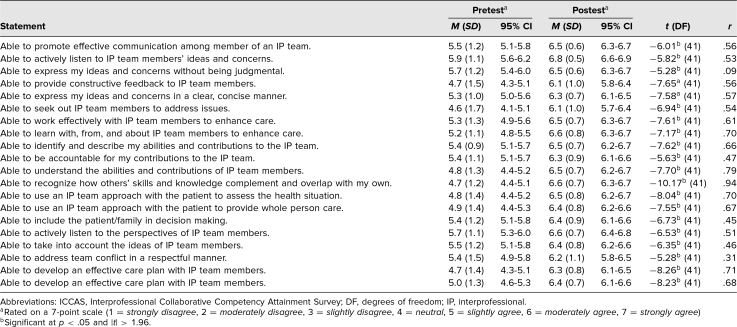
Retrospective Pre- and Postsurvey Participant Responses to ICCAS (*N* = 68)

## Discussion

As health care grows more complex, there is a tendency for health care professionals to become siloed within their respective roles in patient care. Our goal was to create an educational impact in the areas of medication safety and adherence, oral health care, environmental safety, rehabilitation, as well as transitional patient care by developing an IPE simulation. Early IPE integration into educational curricula can lay the foundation for future professionals. However, several barriers exist that make the incorporation of IPE programs challenging.^7,8^ Lack of an integrated IPE curriculum throughout all health care curricula can make scheduling and resultant buy-in from faculty and students challenging.^7^ We navigated this challenge by using a centralized IPE department, as well as our centralized simulation center at our academic institution. The simulated environment created a low-stakes scenario for the learners to perform patient interviews and assessment to enhance real-world applicability to their education. The ICCAS responses postsimulation showed positive outcomes in perceived abilities and increased understanding of the scope of practice of other health care professionals.

All learners volunteered to partake in the simulation, which may have contributed an element of selection bias into responses. However, this simulation did showcase a novel approach to active, experiential learning of HBC, and the results suggested incorporation of this simulation into IPE may be useful for creating strong foundational interprofessional concepts. Our level of learner would also vary between the groups. Moving forward, we felt this overall had little impact, provided the learner had some prior clinical experience as this would embolden them to share their reflections. Our groups would vary in size as well; when there were larger groups of greater than six or more participants, the professions of nursing, physical, and OT would often take the lead in the encounter as they often had more didactic training in home health encounters. These professions would drive the interaction with the SP, and the medicine, pharmacy, and dentistry cohorts would observe and then offer their observations or guidance after the SP interview. All groups were given the opportunity to formulate a strategy prior to the simulation encounter, and the most successful encounters had a designated leader in the group from nursing, PT, or OT. Moving forward, based on student and faculty feedback, the group is transitioning this simulation to a permanent experience integrated into the appropriate course within each health care discipline's curricula. Shifting this simulation activity to a course or experiential education requirement could help ease the burden of recruiting learners to volunteer, as their schedules are often very demanding with significant variabilities, as well as creating protected time for the faculty preceptors.

This simulation could be replicated in academic settings with simulation and interprofessional programs; however, the simulation can be resource intensive. Faculty preceptor involvement was on a volunteer basis; however, their contributions could be recognized through the promotion and tenure process. Our institution has a robust simulation center that also made this possible for implementation. As IPE often presents a number of challenges, the faculty team should carefully consider resource needs, scheduling issues, and the appropriate place within the curriculum.^7^ At our institution, a grant was obtained to fund a simulated apartment, and all secured funding was directed toward the creation of the simulated home environment, including simulation props. Despite the resource-intensive nature of the simulation, we felt this project created significant contributions in the education of HBC and highlighted common pitfalls in postacute care transitions. Moving forward, we plan to transition this simulation to a telemedicine encounter given the rapid deployment of telemedicine into the health care system. Moving to a virtual format will likely ease the transportation restrictions if participants require travel to the staged apartment and may eliminate scheduling hurdles for all participants, as well as requiring fewer resources for the live monitoring of the activity. As we are supported by a centralized simulation center, we do have the availability to perform video monitoring in an off-site apartment to give learners the ability to explore the simulated home for safety hazards.

As incorporating IPE events can be challenging, following the IPE learning model developed by the Health Professions Accreditors Collaborative can assist other institutions with developing a similar activity.^14^ This guidance recommends planning IPE events utilizing the four critical components of rationale, outcomes-based goals, deliberate design, and assessment/evaluation.^14^ Lastly, as we used learners in various stages of their training, the level of learner chosen may also affect the subsequent results. Ideally, the learners should have some clinical exposure prior to the simulation, to better enable them to share their clinical reflections with their coparticipants.

Posthospital transitions are times of medical vulnerability for patients and are an area of active improvement for quality of health care delivery.^12^ Several educational strategies emphasized the importance of improved transitional care. However, transitional care is often underemphasized in health care provider education.^8^ Improved interprofessional collaboration has been theorized to improve the quality of transitional care.^9^ As discharge planning can be an important component of all health care providers' practice, improved confidence in seeking interprofessional opinions and understanding scope of practice will increase further professional collaboration. While the generalizability of self-reported learner confidence is difficult to translate into patient outcomes, we hope to elucidate further potential impacts in improving the quality of transitional care as this project continues to move forward.

The ICCAS is designed to assess the change in self-reported competencies in collaboration among health care students.^13^ The ICCAS is validated and can predict meaningful outcomes with regard to attitudes toward IPE, but it can be difficult to assess if improved attitudes toward collaboration are meaningful in reducing patient harm in the clinical setting.^13^ As such, future plans for this simulation include transitioning to real patient encounters involving interprofessional student teams in rural communities postdischarge. Translating this simulation experience into clinical practice will lead to measurable patient outcomes and move forward into the behavior and results domains of Kirkpatrick's model for learning evaluations.^15^ Several professions are key players in safe transitional practices and discharge planning. With the appropriate resources, this activity can serve as a model for other institutions to incorporate IPE into their graduate and undergraduate health professions curricula.

## Appendices

HBC Simulation Case.docxEnvironment and Equipment.docxPrebrief.docxDebrief.docx
All appendices are peer reviewed as integral parts of the Original Publication.

## References

[1] 65 and older population grows rapidly as baby boomers age. United States Census Bureau. June 25, 2020. Accessed January 9, 2021. https://www.census.gov/newsroom/press-releases/2020/65-older-population-grows.html

[2] Landers S, Madigan E, Leff B, et al. The future of home health care: a strategic framework for optimizing value. *Home Health Care Manag Pract*. 2016;28(4):262–278. 10.1177/1084822316666368 PMC5052697 27746670

[3] Thomé B, Dykes AK, Hallberg IR. Home care with regard to definition, care recipients, content and outcome: systematic literature review. J Clin Nurs. 2003;12(6):860–872. 10.1046/j.1365-2702.2003.00803.x 14632979

[4] Yao NA, Ritchie C, Cornwell T, Leff B. Use of home-based medical care and disparities. J Am Geriatr Soc. 2018;66(9):1716–1720. 10.1111/jgs.15444 30084141

[5] Kramer BJ, Creekmur B, Mitchell MN, Saliba D. Expanding home-based primary care to American Indian reservations and other rural communities: an observational study. J Am Geriatr Soc. 2018;66(4):818–824. 10.1111/jgs.15193 29529341

[6] Karpa K, Graveno M, Brightbill M, et al. Geriatric assessment in the primary care environment: a standardized patient case activity for interprofessional students. MedEdPORTAL. 2019;15:10844. 10.15766/mep_2374-8265.10844 31911935 PMC6944254

[7] Lash DB, Barnett MJ, Parekh N, Shieh A, Louie MC, Tang TTL. Perceived benefits and challenges of interprofessional education based on a multidisciplinary faculty member survey. Am J Pharm Educ. 2014;78(10):180. 10.5688/ajpe7810180 25657367 PMC4315202

[8] Paradis E, Whitehead CR. Beyond the lamppost: a proposal for fourth wave of education and collaboration. Acad Med. 2018;93(10):1457–1463. 10.1097/ACM.0000000000002233 29620672 PMC6159689

[9] Misra S, Teal C, Hatfield C, Major A, Waterhouse A, Gill A. No place like home: the impact of medical students of an interprofessional home visit experience with pharmacy students. MedEdPublish. 2017;6(4):48. 10.15694/mep.2017.000217

[10] Mather M, Jacobsen LA, Pollard KM. Aging in the United States. Population Reference Bureau. December 2015. Accessed January 9, 2021. https://www.prb.org/wp-content/uploads/2016/01/aging-us-population-bulletin-1.pdf

[11] Gadbois EA, Tyler DA, Shield R, et al. Lost in transition: a qualitative study of patients discharged from hospital to skilled nursing facility. J Gen Intern Med. 2019;34(1):102–109. 10.1007/s11606-018-4695-0 30338471 PMC6318170

[12] Little M, Gammack J. Safety first! Transitions from hospital to postacute care. MedEdPORTAL. 2014;10:9789. 10.15766/mep_2374-8265.9789

[13] Archibald D, Trumpower D, MacDonald CJ. Validation of the interprofessional collaborative competence attainment survey (ICCAS). J Interprof Care. 2014;28(6):553–558. 10.3109/13561820.2014.917407 24828620

[14] Barzansky B, Borasky S, Wall JR, Vlasses PH, Zorek JA, Brandt BF; Health Professions Accreditors Collaborative, National Center for Interprofessional Practice and Education. Guidance on Developing Quality Interprofessional Education for the Health Professions. Health Professions Accreditors Collaborative. February 1, 2019. Accessed January 9, 2021. https://healthprofessionsaccreditors.org//wp-content/uploads/2019/02/HPACGuidance02-01-19.pdf

[15] Kirkpatrick DL. Evaluating Training Programs: The Four Levels. 2nd ed. Berrett-Koehler Publishers; 1998.

