# Supplementary material for: Interdisciplinary Education Apartment Simulation (IDEAS) Project: An Interdisciplinary Simulation for Transitional Home Care
Source: MedEdPORTAL. 2021 Feb 26;17:11111. doi: 10.15766/mep_2374-8265.11111 (PMC7908376; doi:10.15766/mep_2374-8265.11111)
Supplement: Supplementary file 1 — HBC Simulation Case.docxEnvironment and Equipment.docxPrebrief.docxDebrief.docx [file mep_2374-8265.11111-s001.zip › A. HBC Simulation Case.docx]

| **Appendix A: Simulation Case**  **SIMULATION CASE TITLE: Interdisciplinary Education Apartment Simulation (IDEAS)**  **AUTHORS: Jenna Sizemore, Gina Baugh, Adam Hoffman, Amy Burt, Kimeran Evans, Amy Summers**  **LEARNER AUDIENCE: Nursing, Occupational Therapy, Physical Therapy, Pharmacy, Medicine and Dentistry students.** | |
| --- | --- |
| **PATIENT NAME: Susie Brokenheart**  **PATIENT AGE: 68**  **CHIEF COMPLAINT: Hospital follow-up visit after coronary artery bypass grafting**  **PHYSICAL SETTING: Patient’s home** | |
|  | |
| **Brief narrative description of case** | The patient is seven days post-hospital discharge after having coronary artery bypass grafting. The patient has a medical history of coronary artery disease, hypertension, atrial fibrillation, and hyperlipidemia.  Educational Objectives:  1. Understand the roles and responsibilities of an interprofessional team with regards to home-based care.  2. Develop an effective care plan using an interprofessional team approach for transitions from acute, hospital-based care to the home setting.  3. Demonstrate the use of effective communication among different members of an interprofessional team in home-based care. |
| **Primary Learning Objectives** | Student Learning Objectives:   1. Demonstrate knowledge of roles and responsibilities of team-based behaviors required for home-based healthcare. 2. Demonstrate knowledge of interprofessional communication required for effective transitions from acute, hospital-based care to the home setting. 3. Identify components of environmental safety and home modifications. |
| **Critical Actions** | 1. Through interprofessional collaboration, prioritize safety concerns and patient’s concerns. 2. Identify physical hazards in the home and address safety concerns with the patient. 3. Identify medication errors and address to prevent harm. 4. Perform a physical assessment of the patient. 5. Perform patient counseling on medications, wound care, assistive devices, and movement restrictions. |
| **Learner Preparation or Prework** | The patient case as a discharge summary will be provided prior to the pre-brief. The learners are given time before the simulation to discuss how they want to approach the patient.  They will be instructed to have a plan in place before seeing the patient. Examples include: Who will conduct the interview, and how each person will contribute to patient assessment and plan. |

| Initial Presentation | | | |
| --- | --- | --- | --- |
| CASE: REASON FOR HOSPITALIZATION AND HOSPITAL COURSE: The patient is  68-year-old female with known HTN who presented with a history of palpitations. The patient underwent a myocardial perfusion scan, which revealed a moderate area of ischemia in the septal wall.  Due to a positive MPS, the patient was taken for left heart catheterization which revealed multi vessel coronary artery disease with significant critical disease of the LAD and diagonal system. The cardiac catheterization revealed an ejection fraction of 55-60%. At that time, it was decided that the patient would benefit from coronary artery bypass grafting. 7 days ago, the patient was taken to the OR. She received endotracheal intubation and general anesthesia. She received an off-pump CABG x3 utilizing the LIMA to LAD, reversed saphenous vein graft to the diagonal, reversed saphenous vein graft to the OM in the third position. The patient also underwent endoscopic vein harvesting to attain the venous conduit. The patient tolerated the procedure well and was transferred to the cardiothoracic unit in stable condition. The patient was extubated later that evening. On postop day 1, the patient was weaned off inotropic agents and her urinary catheter was discontinued. She was transferred to the 10 east telemetry unit on postoperative day 2. On postoperative day 2, the patient developed rapid atrial fibrillation and electrolytes were drawn which revealed that she had suffered from hypokalemia and hypomagnesemia which was replaced. The cardiology service was consulted for atrial fibrillation, however, on postoperative day 3, the patient converted to normal sinus rhythm. The patient was changed to amiodarone p.o. On postoperative day 4, her chest tubes were removed. It is now postop day 6 and the patient is in stable condition. She has remained in normal sinus rhythm and since POD #3 and is ready to be discharged home today. | | | |
| **Initial vital signs** | N/A | | |
| **Overall Setting and Appearance** | The simulation takes place in an apartment or house- not in the traditional simulation center- and is pre-staged with common items found in the home, some of which are deliberately designed to be safety concerns or errors. The realistic home setting aids in environmental fidelity and cements the suspension of disbelief of the learners. The home should be clean and well kept, but with trip hazards (i.e., rugs, animal toys) and environmental problems (i.e., narrow doorways and steep stairs). The home should be fully furnished and the items chosen be consistent with the age, condition, income, and background of the patient. The ideal setting also has a room the students can use to pre-brief and debrief, internet access, and sufficient parking. | | |
| **Confederates (e.g., standardized participants) and their roles in the room at case start** | The standardized patient (SP) should be of appropriate age for the case, e.g. at least 50, gender non-contributory. The SP should be given the case well in advance and be given an opportunity to discuss any questions with the faculty. The SP will need a moulaged bandage representing a sternotomy and should be dressed appropriately for a home interview.  The SP should throughout the encounter move slowly and deliberately, and as if he or she is in pain. They should be confused about the rules of sternal precaution and not understand their medications, interactions, and side effects. Since being home, the patient complains of dizziness and shortness of breath, especially when getting up to go to the bathroom. The patient is confused about what medications to take when and how they are to use the walker and cane to get around. The patient also experiences some bleeding when brushing and flossing their teeth. They also have dry mouth.  Diet consists of:   - 2-4 cans of soda daily - Breakfast: Egg, cheese, and bacon on a bagel, 2 cups of coffee with cream and sugar - Lunch: Turkey and cheese sandwich on a roll, pretzels, and a Mt. Dew - Snack: Potato chips - Dinner: Some type of meat (steak, hamburger, hot dog), potato, and canned vegetable - Desert: Ice cream - Does add salt to foods   Exercise: Very limited due to intermittent low back pain  Social History: Previously went to bingo twice weekly prior to surgery. The patient currently lives alone and does have a dog.  Tobacco: ½ ppd of cigarettes  Alcohol: One beer nightly to relax  Family support: Sister is sometimes available to help. She is 70 years old and only drives during the day. Daughter that lives about an hour away and can help on weekends if needed. When the SP talks about her sister and daughter, the SP becomes emotional, crying if able.  Activities of Daily Living: Independent with self-care tasks, but requires numerous breaks to get through the activities due to pain, fatigue, and shortness of breath. Sometimes she may skip getting dressed and wear her pajamas throughout the day to avoid this. | | |
| **HPI** | Patient had a coronary artery bypass graft 10 days prior. The patient went to the hospital complaining of chest pain and shortness of breath.  The patient was discharged 3 days prior with the following instructions:  The patient is to follow-up with the attending cardiac surgeon in 4 weeks. The patient is to follow-up with the cardiologist also in 4 weeks for additional management of coronary artery disease and cardiac rehab phase 2. The patient is to follow-up with their primary care doctor on an as needed basis.  Activity: The patient is full sternal precautions and will receive cardiac rehab phase 2.  Diet: The patient is a full cardiac diet.  Ambulation: required an assistive device.  Self-care- completes with adapted devices.  Cognitive status: alert and oriented x3 | | |
| **Past Medical/Surgical History** | **Medications** | **Allergies** | **Family History** |
| Status post coronary artery bypass grafting x3 (LIMA harvest)  Coronary artery disease  Hypertension  Atrial Fibrillation  Hyperlipidemia | Furosemide 20 mg tablets 1 p.o. daily  Warfarin 2.5 mg tablets MWF, 3.0 mg tablets TThSatSun  Clopidogrel 75 mg tablets 1 p.o. daily; take this for 1 year following CABG  Docusate 100 mg caplets 1 p.o. b.i.d. available over-the-counter take 1 as needed for constipation  Fish oil oral tablets 1 p.o. daily  Metoprolol 12.5 mg p.o b.i.d.  Multivitamin tablet 1 p.o. daily  Nitroglycerin 0.4 mg tablets 1 sublingually every 15 minutes for 3 total doses as needed for chest pain    Oyxcodone-Acetaminophen 5/325 mg tablets 1-2 tablets p.o. q.4 hours p.r.n. for pain, dispense 50 with no refills  Potassium chloride 10 mEq 1 p.o. b.i.d. with food  Simvastatin 20 mg tablets 1 p.o. nightly  Amiodarone 200 mg daily | No known drug allergies | The standardized patient can report their own family history or make up a report when asked by the learners. |
| **Physical Examination** | | | |
| **General** | Patient presents with fatigue and shortness of breath with movement. Patient’s dressings are soiled. Patient is oriented x3. | | |
| **HEENT** | Normocephalic, Atraumatic. | | |
| **Neck** | No JVD. No thryomegaly. | | |
| **Lungs** | Poor thoracic excursion, no adventitious lung sounds | | |
| **Cardiovascular** | Regular rate and rhythm, Pulses 2+ and symmetric in radial, dorsalis pedis distribution | | |
| **Abdomen** | Soft, non-tender, non-distended, normoactive bowel sounds | | |
| **Neurological** | Alert and oriented x 3, Strength 5/5 in upper/lower extremities, no sensory deficits | | |
| **Skin** | No rashes, well-approximated sternotomy incision without purulence or drainage | | |
| **GU** | Deferred. | | |
| **Psychiatric** | Blunt affect, appropriate eye contact, appropriate hygiene, normal speech patterns, non-pressured speech, no flight of ideas, no suicidal ideation | | |

**Expected changes based on learner actions:**

No changes occur in the case based on learner actions.

**Ideal Scenario Flow:**

The learners knock on the patient’s door. The patient opens the door and invites the students into his/ her apartment. The students introduce themselves and the patient invites the students to sit in the living room to begin their home health visit. The students begin to assess the patient through interviewing and active physical assessment/ screenings. The students focus on medications, environment, wound care, functional status and ability, and patient concerns.

After completing a physical examination and obtaining a history, the providers note that the patient is confused about the medications she is taking, eating a poor diet, smoking, and drinking alcohol and other food/ drinks that interact with her medications. The team discusses her medications and strategies to help her remember to take them as well as instructing her to not smoke, drink alcohol, or grapefruit juice as it has adverse effects when combined with her medications. The patient is taught proper wound care. The wheeled walker and raised toilet seat are adjusted for patient safety. The learners also discuss appropriate precautions (sternal precautions) as per the patient’s diagnosis as well as environmental and routine alterations to increase safety and independence in the home. Learners should note activities of daily living that have been impacted by her surgery such as cleaning, showering, pet care, travel and social activities, cooking, and entertainment and should address these with the patient and/or refer her to another specialty or service that can provide assistance.

After interviewing and assessing the patient, the learners examine the physical residence for safety concerns and correct or discuss those concerns with the patient. Examples include: tripping hazards of throw rugs, dog toys on the floor, furniture arranged in a way that will not permit the patient to access chairs while using assistive devices, narrow doorways, high cabinets/closets, low chairs/couches, and steps.

The learners, as a team, should decide the priority of concerns while focusing on patient safety issues and should take care not to overwhelm the patient with more interventions than she can reasonably achieve. The learners should make a plan as to how, who, and when the other concerns will be addressed.

The team should perform all the above as a single unit, with each discipline interacting with the patient at various points but also collaborating with each other to formulate the best care plan as a whole.

**Anticipated Management Mistakes**

*Provide a list of management errors or difficulties that are commonly encountered when using this simulation case.*

1. Commonly, the learners enter the simulation without a feasible plan or without a clear team leader.
2. The learners often identify and attempt to correct too many problems and overwhelm the patient with instructions.
3. Learners often have gotten “stuck” on one set of errors (i.e., medications) to the detriment of identifying or addressing other errors.
4. Rarely, one discipline has “dominated” the assessment or discussion to the detriment of the team, allowing only focus on their concerns for patient care.
5. Commonly, learners will miss errors not “of their discipline” (e.g. medical students not noticing or addressing an off-balance wheeled walker)
6. Commonly, learners will not address the activities of daily living that impact both the patient’s safety and comfort.
