# Supplementary material for: Interdisciplinary Education Apartment Simulation (IDEAS) Project: An Interdisciplinary Simulation for Transitional Home Care
Source: MedEdPORTAL. 2021 Feb 26;17:11111. doi: 10.15766/mep_2374-8265.11111 (PMC7908376; doi:10.15766/mep_2374-8265.11111)
Supplement: Supplementary file 1 — HBC Simulation Case.docxEnvironment and Equipment.docxPrebrief.docxDebrief.docx [file mep_2374-8265.11111-s001.zip › B. Environment and Equipment.docx]

Appendix B

Environment and Equipment

The simulation takes place in an apartment or house (not in the traditional simulation center) and is pre-staged with common items found in the home, deliberately placed to be unsafe. The realistic home setting aids in environmental fidelity and supports the suspension of disbelief of the learners. The home should be clean and well-kept, but with several trip hazards and navigational barriers. The home should be fully furnished and the items chosen be consistent with the age, condition, income, and background of the patient. The ideal facility also needs a room that the students can use to pre-brief and debrief, internet access, and sufficient parking*.* If unfamiliar with the terminology, please refer to *Rehab Concepts and Discharge Dispositions: Workshop for Medical Students*,^1^ Appendix I for detailed descriptions of devices used.

**Medical Equipment:**

- The medical equipment needs to be unsafe, uneven, and/or at a height which would be difficult for the patient to access them with their current limitations.
- Multiple mobility devices (e.g., wheeled walker, cane) in home
- Raised toilet seat.
- Hand held shower.
- Sock aid, reacher, long handled shoe-horn, and/or long handled sponge are not being used properly or not at all.^15^ Stored under bed or couch.

**Medications:**

- Stored in the bathroom. Alternatively can be stored on a window sill or scattered throughout the home.
- At least 7 or more medications- mixture of prescribed medications and over the counter medications.
- Medication bottles depict that multiple pharmacies are used.
- At least 1 medication is expired.
- At least 1 medication with statins and warfarin to counteract with the grapefruit juice.
- Has a medication organization box, but it is not being utilized.

**Standardized Patient:**

- The patient has an uneven walker or a cane and is not using properly when asked to walk by learners.
- Moulage from a chest surgery with scar and bruising and dressing placement.
- Dressings on patient are soiled.

**Miscellaneous Environmental Details:**

- Dirty dressings are on the coffee table and no sterile bandages are available.
- Food in the house is minimal and not healthy such as chips, salt, soda, candy, beer, wine and grapefruit juice.
- Food on countertop and on table. Salt shaker placed on table with meal plate.
- Dirty dishes and empty beer, wine, or liquor bottles on the countertop and/or visibly in the trash can.
- Patient reports having a pet in the house. Therefore, the environment should have food bowls and pet toys that are hard to reach and are trip hazards.
- Step stool in kitchen to reach high cabinets.
- Throw rugs throughout the house, particularly in the bathroom and kitchen.
- Low or high bed.
- Poor lighting.
- Extension cords laying in the walkways.
- No smoke detectors.
- Mouthwash with alcohol.
- Sunken in couch cushions or unsafe chair which the patient sits in for the majority of the encounter.
- Candy in dishes throughout the house.
- Packs of cigarettes and ash trays around the home.
- A worn toothbrush in the bathroom.

**References**

1. Nonaillada J. Rehab concepts and discharge dispositions: workshop for medical students. *MedEdPORTAL.* 2018;14:10785. <https://doi.org/10.15766/mep_2374-8265.10785>
