# Supplementary material for: Interdisciplinary Education Apartment Simulation (IDEAS) Project: An Interdisciplinary Simulation for Transitional Home Care
Source: MedEdPORTAL. 2021 Feb 26;17:11111. doi: 10.15766/mep_2374-8265.11111 (PMC7908376; doi:10.15766/mep_2374-8265.11111)
Supplement: Supplementary file 1 — HBC Simulation Case.docxEnvironment and Equipment.docxPrebrief.docxDebrief.docx [file mep_2374-8265.11111-s001.zip › C. Prebrief.docx]

Appendix C

IDEAS Prebrief

**Instructions:** The Program Specialist sends the learners an email with the patient’s discharge summary (below), information regarding the time, date and location of the simulation and any additional information needed for the students to access the simulation activity.

The learners receive an email from the STEPS LearningSpace specialist with instructions on how to access LearningSpace to complete the ICCAS survey before exiting the simulation.

The learners will be given 10 minutes to introduce themselves to other disciplines, plan the interview and assessment of the patient. They will choose a leader and discuss the flow of the interview process.

DEPARTMENT OF CARDIAC SURGERY

DISCHARGE SUMMARY

PATIENT NAME: Susie Brokenheart

HOSPITAL NUMBER: 000xxx000

DATE OF BIRTH: 6/10/1952, (or age 68)

ADMISSION DATE: 7 days ago

DISCHARGE DATE: yesterday

ATTENDING PHYSICIAN: Dr. J. Doe, MD.

PRIMARY CARE PROVIDER: SC Blue, MD.

FOLLOW-UP PHYSICIAN: Dr. J. Doe, MD

DATE OF SURGERY: 7 days ago.

DISCHARGE DIAGNOSES:

1. Status post coronary artery bypass grafting x3.

2. Coronary artery disease.

3. Hypertension.

4. Atrial Fibrillation.

5. Hyperlipidemia.

CODE STATUS: The patient is a full code.

DISCHARGE MEDICATIONS:

1. Furosemide 20 mg tablets 1 p.o. daily.

2. Warfarin 2.5 mg tablets MWF, 3.0 mg tablets T,Th,Sat,Sun.

3. Clopidogrel 75 mg tablets 1 p.o. daily

4. Docusate 100 mg caplets 1 p.o. b.i.d. available over-the-counter take 1 as needed for constipation.

5. Fish oil oral tablets 1 p.o. daily.

6. Metoprolol Tartrate 12.5 mg p.o. b.i.d

7. Multivitamin tablet 1 p.o. daily.

8. Nitroglycerin.4 mg tablets 1 sublingually over 15 minutes for 3 doses as needed for chest pain.

9. Oxycodone – Acetaminophen 5/325 mg tablets 1-2 tablets p.o. q.4 hours p.r.n. for pain, dispense 50 with no refills.

10. Potassium chloride 10 mEq 1 p.o. b.i.d. with food.

11. Simvastatin 20 mg tablets 1 p.o. nightly.

12. Amiodarone 200 mg daily

DISCHARGE INSTRUCTIONS:

A. Disposition: The patient is to follow up with Dr. Green in 4 weeks time. The patient is to follow up with Dr. Heart, the cardiologist, also in 4 weeks time for additional management of coronary artery disease and cardiac rehab phase 2. The patient is to follow up with his PCP, Dr. Blue, on a p.r.n. basis

B. Activity: The patient is full sternal precautions and will receive cardiac rehab phase 2.

C. Diet: The patient is a full cardiac diet.

D: Wounds: Daily Dressing changes

REASON FOR HOSPITALIZATION AND HOSPITAL COURSE: The patient is 68-year-old female with known HTN who presented with a history of palpitations. The patient underwent a myocardial perfusion scan which revealed a moderate area of ischemia in the septal wall. Due to a positive MPS, the patient was taken for left heart catheterization which revealed multivessel coronary artery disease with significant critical disease of the LAD and diagonal system. The cardiac catheterization revealed an ejection fraction of 55-60%. At that time, it was decided that the patient would benefit from coronary artery bypass grafting. 7 days prior, the patient was taken to the OR. She received endotracheal intubation and general anesthesia. She received an off-pump CABG x3 utilizing the LIMA to LAD, LIMA graft to the diagonal, reversed saphenous vein graft to the OM in the third position. The patient also underwent endoscopic vein harvesting to attain the venous conduit. The patient tolerated the procedure well and was transferred to the cardiothoracic unit in stable condition. On postop day 1, the patient was weaned off inotropic agents and her Foley catheter was discontinued. She was transferred to the 10 southeast telemetry unit on postoperative day 2. On postoperative day 2, the patient developed atrial fibrillation with RVR and electrolytes were drawn which revealed that she had suffered from hypokalemia and hypomagnesemia which was replaced. The cardiology service was consulted for atrial fibrillation, however, on postoperative day 3, the patient converted to normal sinus rhythm. The patient was changed to amiodarone p.o. On postoperative day 4, her chest tubes were removed. It is now postop day 6 and the patient is in stable condition. She has remained in normal sinus rhythm and since POD #3 and is discharge ready.

CONDITION ON DISCHARGE:

A. Ambulation ability: Partial- requires assist device.

B. Self-care ability: Partial- with adaptive device.

C. Cognitive status: Alert and oriented x3.

Jane Doe, PA-C

Physician Assistant-Certified

WVU Department of Surgery

Dr. J. Doe, MD

Professor

WVU Department of Surgery
