# Supplementary material for: Interdisciplinary Education Apartment Simulation (IDEAS) Project: An Interdisciplinary Simulation for Transitional Home Care
Source: MedEdPORTAL. 2021 Feb 26;17:11111. doi: 10.15766/mep_2374-8265.11111 (PMC7908376; doi:10.15766/mep_2374-8265.11111)
Supplement: Supplementary file 1 — HBC Simulation Case.docxEnvironment and Equipment.docxPrebrief.docxDebrief.docx [file mep_2374-8265.11111-s001.zip › D. Debrief.docx]

**Appendix D**

**Debrief**

1. Overall Description: Structured time for prebriefing and debriefing was built into each simulation session.
   1. Prebriefing: Fifteen minutes prior to each simulation session, one faculty member and one interprofessional simulation specialist prepared the students for the upcoming experience. These sessions included a description of the broad healthcare related goals of this project (Aging in Place and Transitions of Care) as well as the educational goals and objectives for the students. A discussion about the case then followed with the opportunity for students to ask any remaining questions. Finally, faculty and staff left the room and allowed students to develop a plan for the upcoming simulation activity.
   2. Debriefing: Immediately following each simulation session, the students, one faculty member and one interprofessional simulation specialist provided a structured opportunity for session debriefing. These were always the same faculty member and simulation specialist who conducted the prebriefing. These sessions typically ranged from 30 to 60 minutes in length and were an excellent opportunity to enrich the student learning and receive valuable qualitative feedback about the experience.
2. Debriefing Techniques Employed
   1. Open-Ended Questions regarding Session Learning Objectives:
      1. Demonstrate knowledge of roles and responsibilities of team-based behaviors required for home-based healthcare.
         1. Sample questions included:
            1. *What did you learn about the roles of the healthcare professions who participated in this learning experience?*
            2. *What surprised you the most about the role of the healthcare professions who participated in this learning experience?*
      2. Demonstrate knowledge of interprofessional communication required for effective transitions from acute, hospital-based care to the home setting.
         1. Sample questions included:
            1. *What did you learn about the home care setting?*
            2. *What would improve care for patients discharged home following a hospitalization?*
      3. Identify components of environmental safety and home modifications.
         1. Sample questions included:
            1. *What were the primary findings of your environmental assessment?*
            2. *What recommendations would you make to the patient/client regarding home modifications?*
   2. Specific Debriefing Techniques Employed
      1. Plus Delta: This tool provides a simple method for individuals or teams to engage in reflection using a pluses to identify things that worked and delta to identify things that they may change in the future.
         1. Plus questions included:
            1. *What worked well?*
            2. *What will you continue to use and build upon?*
         2. Delta questions included:
            1. *What would you change in your future practice based upon this experience?*
      2. Advocacy/ Inquiry
         1. “I see you performed [a specific action], can you tell me more about what lead you to do that?”
         2. “I noticed the [discipline] took the primary role in the residence, did you decide that as a team and how did it work?”
      3. Debriefing with good judgement (rarely used)
         1. “I saw that you performed [mistake x], and the standard is [correct action y], can you tell me why you did it that way?”
